# Antiparasitic Sesquiterpenes from the Cameroonian Spice *Scleria striatinux* and Preliminary In Vitro and In Silico DMPK Assessment

**DOI:** 10.1007/s13659-017-0125-y

**Published:** 2017-04-18

**Authors:** Kennedy D. Nyongbela, Fidele Ntie-Kang, Thomas R. Hoye, Simon M. N. Efange

**Affiliations:** 1 Pharmacochemistry Research Group, Department of Chemistry, University of Buea, P. O. Box 63, Buea, Cameroon; 2 Department of Chemistry, University of Minnesota, 207 Pleasant Street, SE, Minneapolis, MN USA; 3 Chemical and Bioactivity Information Centre, Department of Chemistry, University of Buea, P. O. Box 63, Buea, Cameroon

**Keywords:** Sesquiterpenes, Pharmacokinetics, Drug metabolism, *Scleria striatinux*

## Abstract

**Abstract:**

The antiparasitic activity and preliminary in vitro and in silico drug metabolism and pharmacokinetic (DMPK) assessment of six isomeric sesquiterpenes (**1**–**6**), isolated from the Cameroonian spice *Scleria striatinux* De Wild (Cyperaceae) is reported. The study was prompted by the observation that two of the compounds (**1** and **2**) exhibited varying levels of antiparasitic activity on *Plasmodium falciparum*, *Trypanosoma brucei rhodesiense, Trypanosoma cruzi* and *Leishmania donovani*. The in silico method employed a total of 46 descriptors, calculated using Schrödinger QikProp software. 18 of these molecular descriptors that are often used to predict DMPK profiles of drug-like molecules have been selected for discussion. In vitro experimental assessment of metabolic stability made use of human liver microsomes, which was used to correlate theoretical predictions with experimental findings. Overall, the test compounds have been found to have acceptable physicochemical properties and fall within the ranges associated with “drug-like” molecules. Moreover, the compounds exhibited minimal degradation in incubations with human liver microsomes. Although some of these compounds have been reported previously (**1**, **2**, **4** and **5**), this is the first report on their antiparasitic activities, as well as assessment of their DMPK profiles. These results have therefore provided a window for further development of this novel class of sesquiterpene molecules as potential antiparasitic drugs.

**Graphical Abstract:**

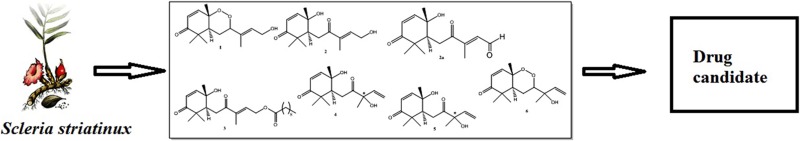

**Electronic supplementary material:**

The online version of this article (doi:10.1007/s13659-017-0125-y) contains supplementary material, which is available to authorized users.

## Introduction

Parasitic diseases, such as malaria and to a lesser extent human African trypanosomiasis (HAT, sleeping sickness) and leishmaniasis, pose an increasing threat to human health and welfare, especially in developing countries [1]. Although effective treatments exist for the clinical management of these diseases, such treatments are costly, marginally effective, and sometimes have unacceptable levels of toxicity. Additionally, there is a steady increase in the resistance of parasites to these drugs. For example, the clinical management of malaria has become more challenging, due to resistance of parasites to drugs such as chloroquine and sulfadoxine–pyremethamine, which were once regarded as the mainstay of malaria chemotherapy [2] and reports of artemisinin resistance of *Plasmodium falciparum* around western Cambodia [3]. Melarsoprol, the only drug that treats *Trypanosoma brucei rhodesiense* and *T. b. gambiense* infections is extremely toxic [4]. The major liability of antimonials, pentamidine and amphotericin B in the treatment of leishmaniasis resides in their safety and cost that takes them out of reach of the local poor.

Natural products (NPs) from plants continue to be one of the promising sources for developing drugs for the treatment of these diseases. [5–7]. For the past 60 years, quinine and its analogues have served as successful drugs for the treatment of malaria, alongside artemisinin (qinghaosu), the main bioactive ingredient of *Artemisia annua*. Thus, drug discovery efforts often resort to NPs as leads or as templates for the synthesis of lead compounds [8]. Unfortunately, many compounds that could serve as leads often fail to enter the market due to poor pharmacokinetic and metabolism profiles. This is mostly due to shortcomings in efficacy, safety and toxicity issues [9]. One approach rapidly gaining support to overcome this setback is the use of in silico (computer-based methods) evaluation of the drug metabolism and pharmacokinetic (DMPK) profiles of these compounds early in the preclinical drug discovery/development pipeline [10–14]. Thus, the early experimental assessment of the pharmacokinetics profiles of drug molecules would ensure that compounds do not fail in the clinical trials, which could have been identified earlier and discarded from the pipeline. In silico approaches, often used for early predictions, are relatively less costly and less time involved, when compared to standard experimental approaches [9, 15]. The accumulation of DMPK data of compounds in pharmaceutical companies has led to the development of in silico models to predict DMPK profiles of newly identified compounds. One way therefore to reduce attrition rates at later stages of drug discovery is to check lead compounds for their absorption, distribution, metabolism, excretion and toxicity (ADMET) properties in the early stages of the drug discovery/development pipeline (Table 1).

A survey of Cameroonian medicinal plants for the evaluation of antimicrobial activity revealed that *Scleria striatinux* De Wild (syn. *S. striatonux, S. woodii*) possessed a very pronounced inhibitory activity [16]. Traditionally the rhizome is used in some parts of Cameroon as a spice. The plant decoction is also administered as a febrifuge. As part of our ongoing antiparasitic drug discovery effort from medicinal plants of Cameroon, we embarked on the phytochemical screening of the rhizome of this plant. Isolation and structure elucidation afforded a new class of isomeric sesquiterpenes, which have been the subject of previous publications [17–19]. These compounds exhibit varying levels of antiparasitic activity. We now report the antiparasitic activity and preliminary in vitro and in silico drug metabolism and pharmacokinetic assessment of the isomeric sesquiterpenes from *Scleria* sp.

## Results and Discussion

**Fig. 1:**
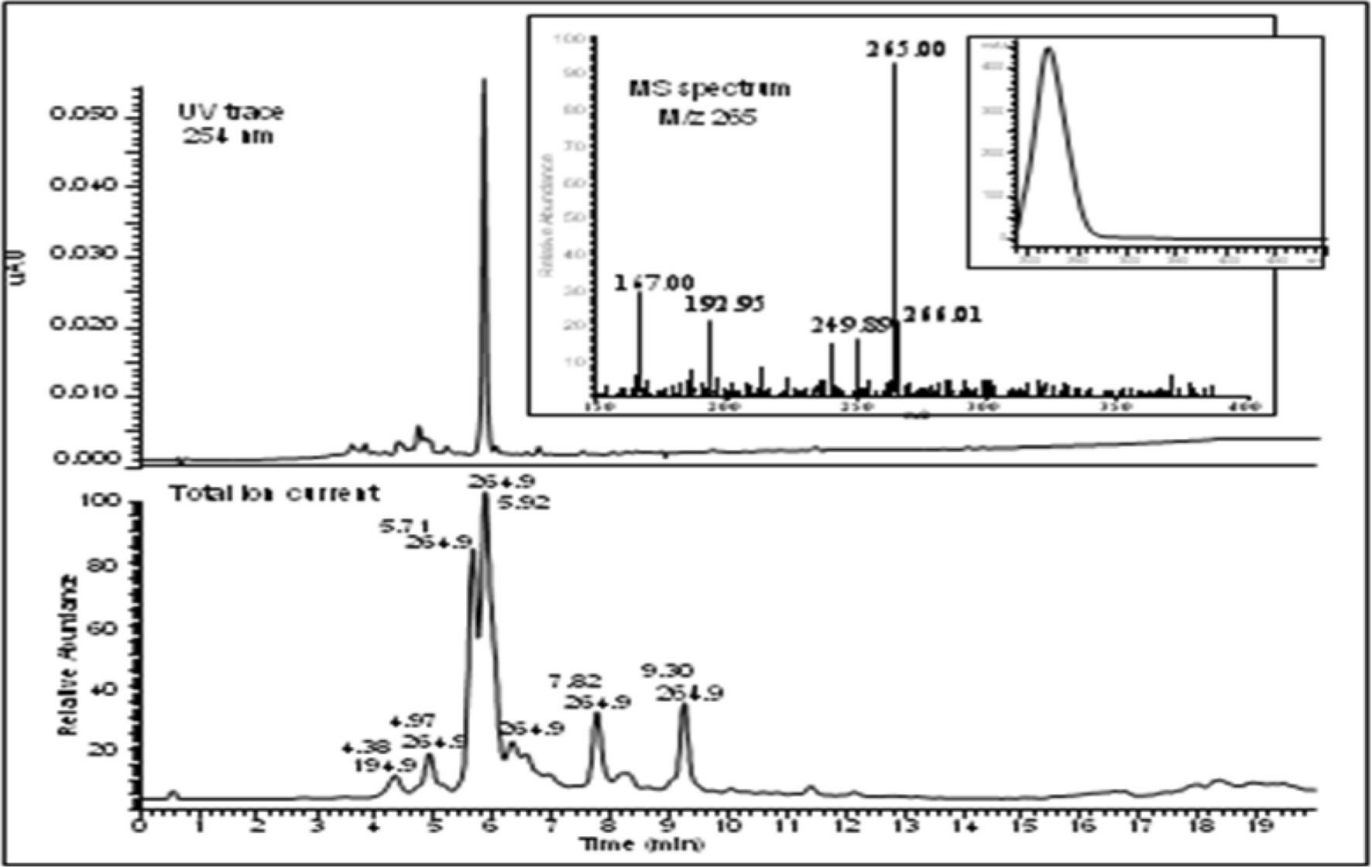
LC/APCI-MS analysis of the active fraction

**Fig. 2:**
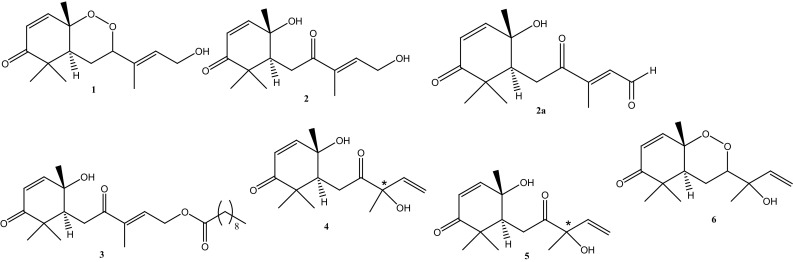
Structures of isolated compounds

Bioassay-guided fractionation of the methylene chloride/methanol (1:1) extract was carried-out on an open silica gel chromatographic column using *n*-hexane, ethyl acetate and methanol in increasing polarity. This afforded several fractions, which were pooled on the basis of their TLC profiles and purified on Sephadex LH-20 using CH_2_Cl_2_ as eluent. HPLC–UV analysis of the identified bioactive fractions from the latter separation procedure, revealed the presence of compounds which display same UV spectra with one maximum around 220 nm (Fig. 1). In the LC/APCI-MS analysis, a base ion recorded at *m/z* 265 for the main peaks suggested the presence of either isomers or compounds with the same base structure (Fig. 1). Semi-preparative HPLC of the above fraction afforded six new sesquiterpene isomers **1–6**. Structure elucidation was achieved on the basis of chemical evidence and extensive spectral studies, using routine 1D- and 2D-NMR spectroscopy. ^1^H NMR spectra revealed four methyl and the presence of vinylic protons in all six compounds. Two of the vinylic protons constituted an isolated α, β-unsaturated ketone (enone) moiety which was suggested from chemical shift values between δ 5.58 and 6.78. ^13^C NMR revealed fifteen carbons with at least one carbonyl moiety in all six compounds (Table 2). From the similarities in both the NMR and MS data for all six compounds, they were characterized as a new class of sesquiterpene diastereoisomers, isolated for the first time from the genus *Scleria* (Fig. 2).

**Table 1:** Selected computed ADMET-related descriptors and their recommended ranges for 95% of known drugs

Property	Description	Recommended range
*S*_mol_	Total solvent-accessible molecular surface, in Å^2^ (probe radius 1.4 Å)	300–1000 Å^2^
ro5	Number of violations of Lipinski’s “Rule of Five” [12, 14]	0
ro3	Number of violations of Jorgensen’s “Rule of Three” [50]	0
*S*_mol,hfob_	Hydrophobic portion of the solvent-accessible molecular surface, in Å^2^ (probe radius 1.4 Å)	0–750 Å^2^
*V*_mol_	Total volume of molecule enclosed by solvent-accessible molecular surface, in Å^3^ (probe radius 1.4 Å)	500–2000 Å^3^
log *S*_wat_	Logarithm of aqueous solubility [47, 49]	−6.0–0.5
log *K*_HSA_	Logarithm of predicted binding constant to human serum albumin [51]	−1.5–1.2
CNS	Predicted central nervous system activity on a −2 (inactive) to +2 (active) scale	−2 (inactive, +2 (active)
log *B/B*	Logarithm of predicted blood/brain barrier partition coefficient [52–54]	−3.0–1.0
*BIP*_caco–2_	Predicted apparent Caco-2 cell membrane permeability, in nm s^−1^ (in Boehringer–Ingelheim scale, [55–57]	<5 low, >100 high
*MDCK*	Predicted apparent Madin-Darby canine kidney cell permeability in nm s^−1^ [56]	<25 poor, >500 great
PHOA	Predicted percentage human oral absorption	>80% high, <25% poor
*Ind*_*c*oh_	Index of cohesion interaction in solids, calculated from the number of hydrogen bond acceptors (HBA), donors (HBD) and the surface area accessible to the solvent, SASA (*S*_mol_) by the relation $Ind_{\text{coh}} = {\text{HBA}} \times \sqrt {\text{HBD}} /S_{\text{mol}}$ [49]	0.0–0.05
Glob	Globularity descriptor, Glob = (4*πr* ^2^)/*S*_mol_, where *r* is the radius of the sphere whose volume is equal to the molecular volume	0.75–0.95
*QP*_*p*olrz_	Predicted polarizability	13.0–70.0
log *HERG*	Predicted IC_50_ value for blockage of HERG K^+^ channels, [33, 34]	Concern <−5
log *K*_p_	Predicted skin permeability [27, 28]	−8.0 to −1.0
*#*Metab	Number of likely metabolic reactions	1–8

**Table 2:** NMR spectral data for compounds **1–6** in CDCl_3_ (500 MHz)

Pos.	1			2			3			4		5		6	
	^1^H NMR δ, mult., J(Hz)	^13^C NMR	HMBC	^1^H NMR	^13^C NMR	HMBC	^1^H NMR	^13^C NMR	HMBC	^1^H NMR	^13^C NMR	^1^H NMR	^13^C NMR	^1^H NMR	^13^C NMR
1	4.26, dd, (5.5)	59.2 (CH_2_)	C-2, C-3	4.48, d, (5.0)	59.2 (CH_2_)	C-3, C-4	4.85, d, (5.0)	61.3 (CH_2_)	C-3′	5.55, d, (16.6) 5.34 d, (11.2)	116.8 (CH_2_)	5.55, d, (17.5) 5.33, d, (10.4)	116.3 (CH_2_)	5.23, d, (10.5) 5.24, d (17.4)	115.7 (CH2)
2	5.75, t, (6.5)	128.6 (CH)	C-1, C-4, C-15	6.78, d, (10.5)	140.8 (CH)	C-4	6.6, t, (4.8)	139.1 (CH)	C-15, C-4	6.02, dd, (11.0, 17.0)	139.6 (CH)	5.99, dd, (11.0, 17.0)	139.3 (CH)	5.89, dd, (10.5, 17.0)	144.3 (CH)
3		135.1 (C)			134.7 (C)			135.3 (C)			80.2 (C)		80.1 (C)		69.4 (C)
4	4.51, dd, (11.2, 2.5)	86.9 (CH)	C-2, C-3, C-15		203.1 (C)			201.6 (C)			212.2 (C = O)		212.2 (C = O)	3.32, dd, (10.5, 11.0)	90.2 (CH)
5a	1.92, ddd, (13, 13, 11)	26.1. (CH_2_)	C-3, C-4, C-6, C-7	2.95, d, (11.0, 3.0)	33.5 (CH_2_)	C-3, C-7, C-11	2.93, dd, (6.0)	34.0 (CH_2_)	C-7, C-11	2.85, m	32.8 (CH2)	2.78, m	32.4 (CH2)	1.27, dd (11.0, 3.0)	
5b	1.66, dt, (13, 3)		C-6, C-7	1.79, d, (13.0, 3.0)		C-3, C-7, C-11				2.76, m		2.65, m		1.52, dd (11.0, 3.0)	
6	2.41, dd, (13.0, 3.3)	49.7 (CH)	C-5, C-7, C-11, C-12, C-13	2.80 dd, (13.0, 3.0)	48.9 (CH)	C-5, C-7, C-10, C-11	2.85, t, (6.0)	49.4 (CH)	C-5, C-7, C-11, C-14	2.85, m	49.0 (CH)	3.01, m	49.2 (CH)	1.89, dd, (10.4, 11.3)	46.4 (CH)
7		79.6 (C)			70.9 (C)			71.1 (C)			71.4 (C)		71.3 (C)		71.9 (C)
8	6.54, d, (10.2)	150.6 (CH)	C-6, C-10	6.78, d, (10.5)	155.3 (CH)	C-6, C-10	6.76, d, (10.2)	155.6 (CH)	C-6, C-7, C-10, C-14	6.80, d (10.3)	155.4 (CH)	6.80, d (10.3)	155.2 (CH)	6.78, d (10.4)	146.9 (CH)
9	5.58d, (10.5)	128.1 (CH)	C-7, C-11	5.84, d, (10.5)	124.9 (CH)	C-7, C-11	5.78 d, (10.2)	125.2 (CH)	C-7, C-11	5.90 d (10.3)	125.6 (CH)	5.90, d (10.3)	125.4 (CH)	6.13, d, (10.4)	126.1
10		203.6 (C)			203.1 (C)			203.2 (C)			202.7 (C = O)		202.7 (C = O)		203.0 (C = O)
11		43.7 (C)			45.2 (C)			45.5 (C)			45.5 (C)		45.2 (C)		45.2 (C)
12	1.05, s	20.8 (CH_3_)	C-6, C-10, C-11, C-13	1.08, s	21.2 (CH_3_)	C-6, C-11, C-13	1.12, s	24.9 (CH_3_)	C-6, C-11, C-13,	1.06 s	25.4 (CH_3_)	1.12, s	25.2 (CH_3_)	1.26, s	21.8 (CH_3_)
13	1.15, s	25.9 (CH_3_)	C-6, C-10, C-11, C-13	1.08, s	24.5 (CH_3_)	C-6, C-11, C-13	1.12, s	14.3 (CH_3_)	C-6, C-11, C-12,	1.06 s	23.1 (CH_3_)	1.06, s	22.9 (CH_3_)	1.26, s	23.8 (CH_3_)
14	1.55, s	21.4 (CH_3_)	C-6, C-7, C-8	1.32, s	22.9 (CH_3_)		1.31, s	23.3 (CH_3_)	C-6	1.34 s	23.1 (CH_3_)	1.34, s	23.2 (CH_3_)	1.35, s	21.5 (CH_3_)
15	1.74, s	13.9 (CH_3_)	C-2, C-3, C-4	1.89, s	11.9 (CH3)		1.81, s	12.5 (CH_3_)	C-2, C-4	1.54 s	25.2 (CH_3_)	1.53, s	25.2 (CH_3_)	1.71, s	23.7 (CH_3_)
1′								173.8 (C)							
2′							2.33 t, (5.0)	34.3 (CH_2_)							
3′							1.25, m	31.8 (CH_2_)							
4′–9′							1.25, m	30.5 (6CH_2_)							
10′							0.82, t	14.3 (CH3)							

### Antiparasitic Activity

The antiplasmodial testing of the extracts was run in duplicate. The method was based upon the [^3^H] hypoxanthine incorporation assay, as described by Desjardins and modified by Ridley [20, 21] and the data fitted to generate IC_50_ and IC_90_ values, corresponding to the concentrations required to cause 50 and 90% inhibition of parasite growth respectively. The *Plasmodium falciparum* clones used for the testing were W-2 and D-6. W-2 strain otherwise known as the Indochina clone is known to be resistant to chloroquine, pyrimethamine, sulfadoxine and quinine. Meanwhile the D-6 otherwise known as the Sierra Leonian clone is resistant to mefloquine but sensitive to the above named drugs. Chloroquine, quinine and artesunate were used as reference drugs. Results of the antimalarial screening for both extracts and compounds are shown in Table 3. The results showed moderate activity of both the CH_2_Cl_2_ and MeOH extracts, with IC_50_ 664 and 894 ng/mL respectively on both strains. For compounds **1–6** that were tested on chloroquine-sensitive (NF54, D6) and chloroquine-resistant (K1, W2) strains of *P. falciparum*, the results demonstrated that these compounds exhibit considerable antiplasmodial activity. The inhibition of parasite growth was similar for both W-2 and D-6 strains. Compounds **1** and **2** showed activity for all four strains suggesting that these compounds may be good leads for the development of novel antimalarial drugs. Parasite inhibition for compounds **3–6** was similar for both D6 and W2. The compounds were inactive on NF54 (IC_50_ > 1000 ng/mL). Compounds **1** and **2** (due to their quantity) were subsequently tested against *T. cruzi*, *L. donovani* and *T. b. rhodensiense*, parasites responsible for Chagas disease, leishmaniasis and human African trypanosomiasis (sleeping sickness) respectively with Benznidazole, Miltefosine and Melarsoprol included as reference drugs. Cytotoxicity was evaluated using HT-29 (human bladder carcinoma) with podophyllotoxin as reference drug. The results found on Table 4 show significant activity on *L. donovani* and *T. b. rhodesiense* for both compounds. Although the compounds were less toxic to human cells than the parasites, their selectivity was considered marginal from the calculated selectivity indexes (SI). Good selectivity was seen on *L. donovani* for compound **1** (11.4). We conclude that these compounds maybe promising targets for the development of drugs against parasitic diseases.

**Table 3:** Antimalarial activity of crude *S. striatinux* extracts and compounds **1**–**6**

Sample	IC (ng/mL)						
	W2^a^		D6^a^		K1^b^	NF54^b^	Cytotoxicity
	IC_50_	IC_90_	IC_50_	IC_90_	IC_50_		
CH_2_Cl_2_ extract	671	1147	664	1043	NT^c^	NT^c^	NT^c^
MeOH extract	804	1402	894	1560	NT^c^	NT^c^	NT^c^
**1**	268	–	195	–	661	304	NT^c^
**2**	354	–	267	–	511	439	NT^c^
**3**	460	–	539	–	–	>1000	NT^c^
**4**	NT^c^	–	NT^c^	–	–	>1000	NT^c^
**5**	NT^c^	–	NT^c^	–	–	>1000	NT^c^
**6**	470	–	483	–	–	>1000	NT^c^
Chloroquine	84	–	3	–	–	2.3	–
Quinine	97	–	27	–	–	–	–
Artesunate	–	–	–	–	–	1.9	–

Results are a mean of 2–3 determinations and individual measurements differed by less 50%

^a^Results obtained from WRAIR

^b^Results obtained from STI

^c^ *NT* not tested

**Table 4:** Antitrypanosomal, antileishmanial, cytotoxicity activities of compounds **1** and **2.** And calculated selective index

Sample	IC_50_ (ng/mL)						
	*T. cruzi* ^a^	SI^b^	*L. donovani* ^a^	SI	*T. b. rhod* ^a^	SI	Cytotoxicity^a^
**1**	22.7	1	2	11.4	6.7	3.4	22.7
**2**	6.6	0.74	0.83	5.9	0.59	8.3	4.9
Benznidazole	0.282	–	–	–	–	–	–
Miltefosine	–	–	0.156	–	–	–	–
Melarsoprol	–	–	–		0.001	–	–
Podophylotoxin	–	–	–	–	–	–	0.007

^a^Results obtained from STI

^b^ *SI* selective index

### Preliminary In Silico and In Vitro, Drug Metabolism and Pharmacokinetic Evaluation

Compounds **1**, **2** and **2a** were selected due to their in vitro data and evaluated for their physicochemical properties and drug metabolism profiles in human liver microsomes. The physicochemical characteristics of the compounds were assessed using a mixture of in silico and experimental techniques as described in the methods section. In vivo predictions of hepatic clearance were based on the assumptions outlined in [22].

### Solubility Measurement

The solubility of the compounds was estimated via the turbidimetric method using standard test buffers (pH 2.0 and pH 6.5) as previously described [23]. The results show that the compounds have good solubility under both test conditions. At pH 2.0, compound **2** was found to have solubility in the range 50–100 µg/mL, whereas at pH 6.5, the solubility exceeded 100 µg/mL. There was no significant change in ionization in the molecule across this pH range. This solubility difference probably reflects subtle solvation changes and indicates that the actual solubility of the compound at pH 6.5 may not be much >100 µg/mL.

### Determination of Partition Coefficient

The partition coefficients of the two compounds were measured via the previously described chromatographic technique [24, 25]. The results obtained indicate that the partition coefficient of compound **2** (LogD_7.4_ = 0.35), is significantly lower than that of compound **1** (LogD_7.4_ = 1.71). The LogD_7.4_ value for compound **2** of 0.35 is towards the lower end of the scale normally associated with “drug-like” molecules. This indicates that the compound may have limited permeability and absorption properties. Results of the physicochemical evaluation are shown on Table 5. For compound **2**, parameters were also calculated for its terminal aldehyde tautomer, **2a**, which is presumed to be the major form of the compound. The overall calculated parameters fall within the ranges associated with “drug-like” molecules.

**Table 5:** Summary of the physicochemical data for **1**, **2** and **2a**

Compound	MW^e^	# H-Bond		PSA (A^2^)^h^	FRB^i^	p*K*a	elogD	Solubility (µg/mL)	
		Don^f^	Acc^g^				pH 7.4	pH 2	pH 6.5
**1**	266.34	1	4	55.8	3	14.17^a^	1.71*	>100	>100
**2**	266.34	2	4	74.6	6	13.73^b^ 13.32^c^	0.35*	50–100	>100
**2a**	266.34	1	4	71.4		13.31^d^			

For compound 2, the terminal alcohol is presumed to be the minor tautomeric form while compound 2a, the terminal aldehyde is presumed to be the major tautomeric form

* Value measured using the chromatographic eLogD technique

^a^This p*K*a value corresponds to the acidic terminal alcohol

^b^This p*K*a value corresponds to the acidic terminal alcohol

^c^This p*K*a value corresponds to the acidic secondary alcohol

^d^This p*K*a value corresponds to the acidic secondary alcohol

^e^Molecular weight

^f^Number of hydrogen bond donors

^g^Number of hydrogen bond acceptors

^h^Polar surface area

^i^Number of free rotating bonds

### Metabolism of Compounds **1** and **2** in Human Liver Microsomes

The objective here was to determine the in vitro metabolic stability of these two compounds using human liver microsomes as a preliminary indication of their likely in vivo metabolic clearance and potential metabolic products. The method used was as outlined in Obach [22]. The results (Table 6) show that the two compounds exhibited minimal degradation when incubated with human liver microsomes and based on the in vitro clearance values, they are predicted to show low-to-intermediate hepatic clearance in vivo. The compounds maybe susceptible to glucuronidation in more complicated test systems based on their structure, since both compounds **1** and **2** contain an α, β-unsaturated ketone moiety with the potential to act as a Michael acceptor (by forming adducts with endogenous nucleophiles such as glutathione). However, this was not investigated and would be the subject of future studies in which we hope to use a simple chemical system (i.e., in aqueous buffer containing free glutathione) or examining the extent of degradation in whole blood.

**Table 6:** Calculated metabolic parameters for compounds **1** and **2** based on NADPH dependent degradation profiles in human liver microsomes

Compound	In vitro *CL*_int_ (µL/min/mg protein)	Microsome predicted *CL*_int_ (mL/min/kg)	Microsome predicted *E*_*H*_	Metabolites detected
**1**	12.3	14.3	0.41	None detected
**2**	7.4	8.6	0.29	None detected

The study which has provided preliminary information regarding physicochemical properties and metabolic characteristics of the test compounds, show that, overall, the calculated parameters for these compounds fall within the ranges associated with “drug-like” molecules. One setback for these compounds is the presence of at least one α, β-unsaturated carbonyl moiety, which exposes them to reactivity with endogenous nucleophiles such a glutathione in a Michael-type reaction.

### Oral Bioavailability Prediction Using Lipinski’s “Rule of Five” and Jorgensen’s “Rule of Three”

The results for 18 selected computed molecular descriptors often used to predict DMPK profiles of drug-like molecules have been shown in Table 7. Absorption depends on the solubility and permeability of the compound, as well as interactions with transporters and metabolizing enzymes in the gut wall. Apart from compounds **3** and **6**, all the other compounds showed predicted percentage human oral absorption (PHOA) parameters >80%. This was in agreement with the #star parameter (for all the tested compounds, #star = 0, except for compounds **3** and **6** with respective #star values of 1 and 2). In addition, compound **3** violates both the Lipinski’s “rule of five” (ro5) and Jorgensen’s “rule of three” (ro3) for the prediction of oral bioavailability, which is in agreement with the PHOA predictions. The predicted poor PHOA of compound **3** could also be explained by its low water solubility, which is >3 log values lower than those of all the other tested compounds (Table 7). The violations of the #star parameter for compound **6**, could be explained by its exceptionally low predicted Caco-2 permeability, when compared with the other tested compounds, even though this compound did not violate the ro3.

**Table 7:** Summary of the predicted physicochemical parameters used to access the pharmacokinetic properties of compounds **1**–**6**

Compound	#Stars^a^	Ro5^b^	LogB/B^c^	BIP_caco-2_ (nm s^−1^)^d^	*S*_mol_ (Å^2^)^e^	*S*_mol,hfob_ (Å^2^)^f^	*V*_mol_ (Å^3^)^g^	Log*S*_wat_ (S in mol L^−1^)^h^	Log*K*_HSA_^i^
**1**	0	0	−0.645	723.78	510.17	313.36	896.93	−2.98	−0.213
**2**	0	0	−0.799	620.57	483.87	281.25	882.16	−2.11	−0.353
**3**	1	1	−1.587	813.61	831.10	640.65	1504.62	−6.56	0.753
**4**	0	0	−0.522	1332.91	485.30	313.04	887.04	−2.36	−0.207
**5**	0	0	−0.694	789.50	478.06	282.08	879.54	−2.24	−0.219
**6**	2	0	−0.603	134.86	479.24	271.10	875.88	−2.84	−0.244

	Ro3^j^	CNS^k^	MDCK^l^	PHOA^m^	Glob^n^	QP_polrz_ (Å^3^)^o^	LogHERG^p^	Log*K*_p_^q^	#Metab^r^
**1**	0	0	348.82	87.77	0.88	28.55	−3.81	−3.17	3
**2**	0	−1	295.38	84.87	0.92	26.02	−3.19	−3.02	4
**3**	1	−2	395.84	67.59	0.90	16.52	−2.26	−3.60	1
**4**	0	0	674.91	94.42	0.92	26.26	−3.21	−2.35	3
**5**	0	0	383.18	89.16	0.93	25.96	−3.07	−2.80	3
**6**	0	−1	72.16	78.04	0.92	28.32	−1.31	−3.53	1

^a^Number of times a computed property falls outside the acceptable range for 95% if drugs

^b^Number of violations of Lipinski’s “Rule of Five”

^c^Logarithm of predicted blood/brain barrier partition coefficient (range for 95% of drugs: −3.0–1.0)

^d^Predicted apparent Caco-2 cell membrane permeability in Boehringer–Ingelheim scale, in nm/s (range for 95% of drugs: <5 low, >500 high)

^e^Total solvent-accessible molecular surface, in Å^2^ (probe radius 1.4 Å) (range for 95% of drugs: 300–1000 Å^2^)

^f^Hydrophobic portion of the solvent-accessible molecular surface, in Å^2^ (probe radius 1.4 Å) (range for 95% of drugs: 0–750 (Å^2^)

^g^Total volume of molecule enclosed by solvent-accessible molecular surface, in Å^3^ (probe radius 1.4 Å) (range for 95% of drugs: 500–2000 Å^3^)

^h^Logarithm of aqueous solubility (range for 95% of drugs: −6.0–0.5)

^i^Logarithm of predicted binding constant to human serum albumin (range for 95% of drugs: −1.5–1.5)

^j^Number of violations of Jorgensen’s “Rule of Three”

^k^Predicted activity in the central nervous system (−2 = inactive, +2 = active)

^l^Predicted apparent MDCK cell permeability in nm/sec (<25 poor, >500 great)

^m^Percentage human oral absorption (>80% is high and <25% is poor for 95% of drugs)

^n^Globularity descriptor (0.75–0.95 for 95% of drugs)

^o^Predicted polarizability (13.0–70.0 for 95% of drugs)

^p^Predicted IC_50_ value for blockage of HERG K^+^ channels (concern <−5)

^q^Predicted skin permeability (−8.0 to −1.0 for 95% of drugs)

^r^Number of likely metabolic reactions (range for 95% of drugs: 1–8)

### QikProp Predictions

The blood/brain partition coefficients (log B/B) were computed and used to predict access to the central nervous system (CNS). It was observed that the predicted CNS activities of all tested compounds were quite low, in agreement with the moderate blood/brain barrier (BBB) coefficients. Oral absorption estimates are often done using Madin-Darby Canine Kidney (MDCK) monolayers. The reason is that such cells do express transporter proteins, but only express very low levels of metabolizing enzymes [26]. MDCK cells are also used as an additional criterion to predict BBB penetration. This could explain why estimated MDCK cell permeability could be considered to be a good mimic for the BBB (for non-active transport). Even though all MDCK permeability values fall within the required range for 95% of known drugs, that of compound **6** was quite low, when compared with the rest of the compounds (Table 7).

The efficiency of a drug is often affected by the degree to which it binds to blood plasma proteins. This is because the binding of drugs to plasma proteins (like human serum albumin, lipoprotein, glycoprotein, α, β, and γ globulins) greatly reduces the quantity of the drug in general blood circulation and hence the less bound a drug is, the more efficiently it can cross cell membranes or diffuse. The predicted plasma-protein binding has been estimated by the prediction of binding to human serum albumin; the $\log K_{\text{HSA}}$ parameter (the recommended range for 95% of known drugs is −1.5 to 1.5). This predicted parameter fall within the recommended range for all six tested compounds, as well as those of the predicted skin permeability parameter [27, 28].

Potential toxicities of the tested compounds were predicted by the blockage of human ether-a-go-go related gene (HERG) potassium ion (K^+^) channels, modeled using the logHERG parameter. HERG is known to encode a K^+^ channel that is implicated in the fatal arrhythmia known as *torsade de pointes* or the long QT syndrome [29]. The most important known contribution of the HERG K^+^ channel is its contribution to the electrical activity of the heart that coordinates the heart’s beating. This appears to be the molecular target responsible for the cardiac toxicity of a wide range of therapeutic drugs [30]. HERG is also known to be associated with modulating the functions of some cells of the nervous system and with establishing and maintaining cancer-like features in leukemic cells [31]. HERG K^+^ channel blockers are therefore potentially toxic, hence predicted IC_50_ values could provide reasonable predictions for cardiac toxicity of drugs in the early stages of drug discovery [32]. The predicted logHERG values for all the tested compounds were >−5, which is within the acceptable range [33, 34]. In addition, all the tested compounds were predicted to undergo a maximum of 4 metabolic reactions, which is within the acceptable range for "drug-like" compounds.

## Experimental Section

### General Experimental Procedures

Liquid chromatography was performed with a HPLC system equipped with a binary pump and photodiode array high speed spectrophotometric detector and autosampler, all controlled by the Agilent Chemstation software (Agilent, Palo Alto, CA, USA). A Waters Symmetry C18 (15 mm × 4.6 mm i.d.; 5 μm) column was used for the separation with MeCN–H_2_O (0.1% formic acid) as mobile phase; the separation employed a stepwise gradient that began with 5:95 (MeCN:H_2_O), changed to 40:60 after 10 min and ended with 75:25 (MeCN:H_2_O). The total run time was 25 min. The column was washed with 100% (MeCN) during 5 min. The flow rate was maintained at 1 mL/min. APCI-MS detection was achieved in negative mode on a Finnigan (San Jose, CA, USA) model LCQ ion trap spectrometer equipped with a Finnigan APCI source. The APCI parameters were as follows: heated capillary temperature, 150 °C; vaporizer temperature, 360 °C; discharge current, 5.0 μA; nebulisation gas (nitrogen), 90 psi. In source collision-induced dissociation of 15 eV was used. TOF–MS experiments for accurate mass measurements (resolution of 10,000 FWHM) were conducted on a TOF LCT mass spectrometer (Waters, Manchester, UK). The ESI conditions were as follows: capillary voltage, 2800 V; source temperature, 120 °C; desolvation temperature, 200 °C; cone voltage, 40 V; desolvation gas flow, 600 L/h. MS scan time: 1 + 0.1 s interscan delay. Sulfadimetoxine ([M–H]^−^ 309.0658) was used as lock mass calibrant (Aldrich, Buchs, Switzerland). ^1^H- and ^13^C-NMR spectra were obtained in CDCl_3_ with a Varian Inova Unity 500 spectrometer (499.87 and 125.70 MHz, respectively). Complete assignment was achieved on the basis of 2D experiments (COSY, HSQC, HMBC) using Varian VNMR software.

### Plant Material and Documentation

Samples of *S. striatinux* rhizomes were harvested in Oku in the North West Region of Cameroon by Dr Claire Wirmum. The plant was identified with the collaboration of botanists at the Limbe Botanic Garden and the National Herbarium, Yaoundé, Cameroon. A voucher specimen (No. 32235/HNC) has been deposited at the National Herbarium, Yaoundé, Cameroon.

### Extraction and Isolation

The air-dried rhizomes of *S. striatinux* (10 kg) were ground to powder and extracted with CH_2_Cl_2_/MeOH (1:1) for 2 days to afford 450 g of crude extract. This extract was fractionated chromatographically in portions of 150 g. Fractions of 500 mL were collected from an open silica gel column using selected combinations of *n*-hexane, EtOAc and MeOH as mobile phase. The solvent was removed by evaporation on a rotary evaporator. Fractions were then combined on the basis of TLC profiles. Fractions that eluted with *n*-hexane/EtOAc (6:4) were combined, tested in vitro and found to display moderate antiplasmodial activity. Consequently, this combined fraction was further purified via gel permeation on Sephadex LH-20. Analytical HPLC of the above active subfractions using H_2_O (0.1% formic acid)/CH_3_CN (0.1% formic acid) as mobile phase on a symmetry C18 4.6 × 250 mm column showed similarities in their chromatograms. Compounds **1** (23.1 mg) and **2** (14.7 mg) were collected after semi-preparative HPLC (Shimadzu LC-8A) on an XTerra^®^ Prep. MS C18 OBD™ (150 mm × 19 mm i.d., 5 μm) column. H_2_O (0.1% formic acid) and MeCN (0.1% formic acid) were used as mobile phases, beginning from 10 to 25% of MeCN. The total run time was 50 min. For each run, 400 μL (27.5 mg) was injected at a flow rate of 20 mL/min.

### Antiparasitic Testing

The crude *S. striatinux* extracts (MeOH and CH_2_Cl_2_) were tested at the Walter Reed Army Institute of Research (WRAIR), Washington. DC. Compounds **1–6** were tested at the Swiss Tropical Institute (STI), Basel, Switzerland. The method used for antiplasmodial testing, was based upon [^3^H] hypoxanthine incorporation assay, previously described in the literature [20, 21]. Both chloroquine-sensitive (NF54, D6) and chloroquine-resistant (K1, W2) strains and chloroquine, quinine and artesunate used as positive references. *L. donovani* MHOM-ET-67/L82 (obtained from the spleen of an infected hamster and grown in axenic cultures) was used for the antileishmanial testing while *T. brucei* squib 427 strain (suramin-sensitive) was used for the antitrypanosomal testing. Cytotoxicity test was done using HT-29 (human bladder carcinoma), with podophyllotoxin included as reference drug at a concentration of 0.1 μg/mL.

## Experimental Pharmacokinetic Profiling

### In Vitro Determination of Physicochemical Properties

The kinetic solubility of test compounds was examined by spiking compound in DMSO into either pH 6.5 phosphate buffer or 0.01 M HCl (approximately pH 2.0) with the final DMSO concentration being 1%. Samples were then analysed via nephelometry to determine a solubility range [23]. Effective log D values were measured using a chromatographic method employing a SUPELCOSIL LC-ABZ column using an octanol saturated mobile phase at pH 7.4 [23]. Theoretical physicochemical values were determined using the ACD logD suite of software programs. The molecular weight (MW) should ideally be ≤500 for good membrane permeability, while the hydrogen bond donor/acceptor properties should be respectively ≤5 and ≤10 respectively [24, 25]. The number of single bonds that are not in a ring or constrained system and are not bonded to a hydrogen atom (often referred to as the number of rotatable single bonds) should be ≤10 for acceptable oral bioavailability [26]. The polar surface area (PSA) was calculated using a simplified 2-dimensional modeling approach, which has been validated against a more sophisticated 3-dimentional modeling strategy. For acceptable oral drug absorption and membrane permeability, the PSA value should be ≤120 Å^2^. The basic physicochemical measure of acidity of a compound (p*K*a) only indicate whether ionization is likely to be relevant at physiological conditions (pH = 7.4). The 1-octanol/water partition coefficient were calculated for ionisable compounds. The values were calculated on the basis of log P and p*K*a values. Log D values are quoted with respect to a relevant pH, thereby providing a partition coefficient profile under physiologically relevant conditions.

### Metabolic Stability

Test compounds (2 µM) were incubated at 37 °C with human liver microsomes [purchased from BD Gentest (Woburn, MA) using microsome lot # 41207]. The reaction was initiated by the addition of an NADPH-regenerating system (i.e. NADPH is the cofactor required for CYP450-mediated metabolism) and quenched at various time points over the incubation period by the addition of acetonitrile. Additional samples co-activated by NADPH and UDPGA (the co-factor for glucoronidation), were included in the incubation for the qualitative assessment of the potential for glucoronide formation. The relative loss of parent compound and formation of metabolic products was determined using a single quadrupole LCMS instrument (Waters, Manchester, UK).

Test compound concentration *versus* time data was fitted to an exponential decay function to determine the first-order rate constant for substrate depletion (*k*). Each substrate depletion rate constant was then used to calculate: (1) an initial in vitro clearance value (in vitro, *CL*_int,in vitro_); (2) an intrinsic clearance value (*CL*_int_); and (3) a hepatic extraction ratio (*E*_*H*_)

1 $$CL_{{{\text{int,}}\;{\text{in}}\;{\text{vitro}}}} = \frac{k}{{{\text{microsomal}}\;{\text{protein}}\;{\text{content}}\; ( 0. 4\;{\text{mg}}\;{\text{protein/mL)}}}}$$

2 $$CL_{\text{int}} = CL_{{{\text{int,}}\;{\text{in}}\;{\text{vitro}}}} \times \frac{{{\text{liver}}\;{\text{mass}}\; ( {\text{g)}}}}{{{\text{body}}\;{\text{weight}}\; ( {\text{kg)}}}} \times \frac{{ 4 5\;{\text{mg}}\;{\text{microsomal}}\;{\text{protein}}}}{{{\text{g}}\;{\text{liver}}\;{\text{mass}}}}$$

3 $$E_{H} = \frac{{CL_{\text{int}} }}{{Q + CL_{\text{int}} }}$$

For Eqs. (2) and (3), the following scaling parameters were assumed: hepatic flow rate (*Q*) = 20.7 mL/min/kg; liver mass = 25.7 g liver/kg body mass; microsomal content = 45 microsomal protein/g liver mass.

### Molecular Modelling

All molecular modelling was carried out on a Linux workstation with a 3.5 GHz Intel Core2 Duo processor. Sketching of the 3D structures of the compounds was done using the builder module of the MOE software (CCG, Montreal). Energy minimisation was subsequently carried out using a previously described protocol [35–39], using the MMFF94 forcefield [40], reaching a gradient of 0.01 kcal/mol. The energy minimized structures were then saved (in mol2 format). These were initially treated with LigPrep [41], implemented on the graphical user interface of the Maestro software package [42], using the Optimized Potentials for Liquid Simulations (OPLS) forcefield [43–45]. The protonation states at biologically relevant pH were correctly assigned. This involves the following; disconnection of group I metals in simple salts, deprotonation of strong acids, protonation of strong bases and addition of topological duplicates and explicit hydrogens.

### In Silico Assessment of Pharmacokinetic Profiles

A total of 46 ADMET-related molecular descriptors were calculated by using the QikProp program [46], which was running in normal mode. The pharmacokinetic profiles of the test compounds were evaluated by using an overall ADME-compliance score—drug-likeness parameter (indicated by #stars). This parameter is an indicator of the number of property descriptors computed by QikProp that fall outside the optimum range of values for 95% of known drugs. The methods used and implemented in this program were developed by Jorgensen and Duffy [47–49]. A selection of 18 computed ADMET molecular descriptors that are often used to predict DMPK profiles of drug-like molecules are shown in Table 1 (together with their recommended ranges for 95% of known drugs). In selecting the descriptors, we ensured that at least one descriptor directly related to absorption, distribution, metabolism, elimination and toxicity was included on the list.

## Conclusions

The antiparasitic and DMPK properties of six (06) isomeric sesquiterpenes isolated from *Scleria striatinux*, a medicinal spice of Cameroonian origin, have been evaluated using a mixture of in silico and experimental techniques. Overall, the tested compounds have been found to have acceptable physicochemical properties, but outstanding amongst them are the sesquiterpenes okundoperoxide (**1**) and sclerienone C (**2**). Compound **1** has a good solubility profile, moderate 1-octanol/water partition coefficient and acceptable in silico pharmacokinetic properties, while compound **2** has very similar characteristics to compound **1**, although with less optimal parameters for 1-octanol/water partition coefficient, the polar surface area (PSA) and the number of rotatable bonds. This suggests that compound **1** may have slightly better permeability properties than compound **2**. The DMPK parameters of compounds **3–6** were only modelled using in silico methods. The results revealed that compounds **4** and **5** are as promising as compounds **1** and **2**. Only compounds **3** and **6** have physicochemical properties which fall out of the acceptable range for 95% of known drugs. The studies have demonstrated that compounds **1** and **2** could be two potential lead compounds. These results have thus provided a window for their exploitation as drug candidates against parasitic diseases such as malaria, leishmaniasis and trypanosomiasis that continue to exert a negative toll on our population.

## Electronic supplementary material

Below is the link to the electronic supplementary material.

Supplementary material 1 (DOCX 34 kb)
