# Supplementary material for: Antiparasitic Sesquiterpenes from the Cameroonian Spice Scleria striatinux and Preliminary In Vitro and In Silico DMPK Assessment
Source: Nat Prod Bioprospect. 2017 Apr 18;7(3):235–47. doi: 10.1007/s13659-017-0125-y (PMC5481270; doi:10.1007/s13659-017-0125-y)
Supplement: Supplementary file 1 — Supplementary material 1 (DOCX 34 kb) [file 13659_2017_125_MOESM1_ESM.docx]

**Antiparasitic sesquiterpenes from the Cameroonian spice *Scleria striatinux* and preliminary *in vitro* and *in silico* DMPK assessment.**

**Kennedy D. Nyongbela^1,2,3*^, Fidele Ntie-Kang^2^, Thomas R. Hoye^3^, Simon MN Efange^1^**

***^1^*** *Pharmacochemistry Research Group, Department of Chemistry, University of Buea, P. O. Box 63, Buea, Cameroon. Email: knyongbela@gmail.com*

*^2^ Chemical and Bioactivity Information centre, Department of Chemistry, University of Buea,*

*P. O. Box 63, Buea, Cameroon.*

***^3^*** *Department of Chemistry, University of Minnesota, 207 Pleasant Street, SE, Minneapolis, Minnesota, USA.*

***** To Whom Correspondences should be addressed: knyongbela@gmail.com (KDN)

**Supplementary Data**

**Physicochemical data of compounds**

**Numbering of Compounds**

**Compound 1**

Yellow oil; MF: C_15_H_22_O_4_; [α]_D_ + 72.9 (c 1.6g/100mL, CDCl_3_, 25 ^0^C)

IR (cm^-1^): 3477 (OH), 2989 (C_sp3_H), 2937 C_sp3_H), 2879 (C_sp3_H) and 1674 (C=O)

HR-MS: 289.1402 (Calc. 289.1410) [M+Na]^+^

^1^H NMR: (500 MHz, CDCl_3_): 4.26 (2H, dd, H-1), 5.75 (1H, t, H-2), 4.56 (1H, dd, H-4), 1.96 (1H, dd, H-5a), 1.70 (1H, dt, H-5b), 2.45 (1H, dd, H-6), 6.73 (1H, d, H-8), 5.94 (1H, d, H-9), 1.09 (6H, s, H-12), 1.19 (6H, s, H-13), 1.59 (3H, s, H-14), 1.70 (3H, s, H-15), 1.36 (OH)

^13^C NMR: (500 MHz, CDCl_3_): 59.0 (C-1), 128.3 (C-2), 135.0 (C-3), 86.4 (C-4), 24.7 (C-5), 49.3 (C-6), 79.4 (C-7), 150.3 (C-8), 127.9 (C-9), 203.2 (C-10), 43.5 (C-11), 20.5 (C-12), 26.0 (C-13), 21.1 (C-14), 13.8 (C-15).

**Compound 2**

Light orange oil; MF: C_15_H_22_O_4_

HR-MS (ESI-TOF): 289.1402 [M+Na]^+^, 248 [M-H_2_O]^-^

^1^H NMR: (500 MHz, CDCl_3_): 4.49 (2H, d, H-1), 6.80 (1H, t, H-2), 2.94 (1H, dd, H-5a), 3.00 (1H, dd, H-5b), 2.81 (1H, dd, H-6), 6.78 (1H, d, H-8), 5.88 (1H, d, H-9), 1.07 (3H, s, H-12), 1.09 (3H, s,H-13), 1.34 (3H, s, H-14), 1.82 (3H, s, H-15)

^13^C NMR: (500 MHz, CDCl_3_): 60.3 (C-1), 141.0 (C-2), 137.0 (C-3), 201.8 (C-4), 33.8 (C-5), 49.8 (C-6), 71.1 (C-7), 155.6 (C-8), 125.2 (C-9), 203.2 (C-10), 45.4 (C-11), 21.5 (C-12), 24.9 (C-13), 23.3 (C-14), 12.3 (C-15).

**Compound 3**

Yellow oil; MF: C_24_H_38_O_5_

^1^H NMR: (500 MHz, CDCl_3_): 4.85 (2H, d, H-1), 6.60 (1H, t, H-2), 2.93 (2H, dd, H-5), 2.85 (1H, t, H-6), 6.76 (1H, d, H-8), 5.78 (1H, d, H-9), 1.12 (3H, s, H-12), 1.12 (3H, s, H-13), 1.31 (3H, s, H-14), 1.81 (3H, s, H-15), 2.33 (2H, t, H-2’), 1.25 (10H, m, H-4’-H9’), 0.82 (3H, t, H-10’)

^13^C NMR: (500 MHz, CDCl_3_): 61.3 (C-1), 139.1 (C-2), 135.3 (C-3), 201.6 (C-4), 34.0 (C-5), 49.4 (C-6), 71.1 (C-7), 155.6 (C-8), 125.2 (C-9), 203.4 (C-10), 45.5 (C-11), 24.9 (C-12), 23.3 (C-13), 14.3 (C-14), 12.5 (C-15), 173.8 (C-1’), 34.3 (C-2’), 14.3 (C-10’)

**Compound 4**

White oil; MF: C_15_H_22_O_4_; [α]_D_ +114.3 (c 8.2, MeOH, 25 ^0^C); UV (MeOH) λ_max_ nm (log ε): 285 (5.5); APCI-MS (neg. ion mode): 265 [M-H]^-^, 250, 193, 167

HR-MS (neg. ion mode): 266.1518 [Calc. 265 1440)

^1^H NMR: (500 MHz, CDCl_3_): 5.55 (1H, d, H-1a), 5.34 (1H, d, H-1b), 6.02 (1H, d, H-2), 2.85 (2H, m, H-5a), 2.76 (2H, m, H-5a), 2.85 (1H, m, H-6), 6.80 (1H, d, H-8), 5.90 (1H, d, H-9), 1.06 (3H, s, H-12), 1.06 (3H, s, H-13), 1.34 (3H, s, H-14), 1.54 (3H, s, H-15)

^13^C NMR: (500 MHz, CDCl_3_): 116.8 (C-1), 139.6 (C-2), 80.2 (C-3), 212.2 (C-4), 32.8 (C-5), 49.0 (C-6), 71,4 (C-7), 155.4 (C-8), 125.6 (C-9), 202.7 (C-10), 45.5 (C-11), 25.1 (C-12), 21.4 (C-13), 23.1(C-14), 25.2 (C-15).

**Compound 5**

White oil; MF: C_15_H_22_O_4_; [α]_D_ - 49.5 (c 10.0, MeOH, 25 ^0^C)

UV (MeOH) λ_max_ nm (log ε): 285 (5.5)

APCI-MS (neg. ion mode): 265 [M-H]^-^, 250, 193, 167

HR-MS (neg. ion mode): 266.1518 [Calc. 265 1440)

^1^H NMR: (500 MHz, CDCl_3_): 5.55 (1H, d, H-1a), 5.33 (1H, d, H-1b), 5.99 (1H, d, H-2), 2.78 (2H, m, H-5a), 2.65 (2H, m, H-5b), 3.01 (1H, m, H-6), 6.80 (1H, d, H-8), 5.90 (1H, d, H-9), 1.12 (3H, s, H-12), 1.06 (3H, s, H-13), 1.34 (3H, s, H-14), 1.53 (3H, s, H-15)

^13^C NMR: (500 MHz, CDCl_3_): 116.3 (C-1), 139.3 (C-2), 80.1 (C-3), 212.2 (C-4), 32.4 (C-5), 49.2 (C-6), 71,3 (C-7), 155.2 (C-8), 125.4 (C-9), 202.7 (C-10), 45.2 (C-11), 25.2 (C-12), 22.9 (C-13), 23.2 (C-14), 25.2 (C-15)

**Compound 6**

White oil; MF: C_15_H_21_O_4_

HR-MS (ESI-TOF) negative mode: 289.1193 (calc. 289.1232) [M+Na]^+^

^1^H NMR: (δ, Mult. 500 MHz, CDCl_3_): 5.23 (1H, d, H-1a), 5.24 (1H, d, H-1b)

5.89 (1H, dd, H-2), 3.32 (1H, dd, H-4), 1.27 (1H, dd, H-5a), 1.52 (1H, dd, H-5b), 1.89 (1H, dd, H-6), 6.78 (1H, d, H-8), 6.13 (1H, d, H-9), 1.26 (3H, s, Me-12), 1.26 (3H, s, Me-13), 1.35 (3H, s, Me-14), 1.71 (3H, s, Me-15).

^13^C NMR: (500 MHz, CDCl_3_): 115.7 (C-1), 144.3 (C-2), 69.4 (C-3), 90.2 (C-4), 25.1 (C-5), 46.4 (C-6), 71.9 (C-7), 146.9 (C-8), 126.1 (C-9), 203.0 (C-10), 45.2 (C-11), 21.8 (C-12), 23.8 (C-13), 21.5 (C-14), 23.7 (C-15)

**Table S1:** NMR spectral data for compounds **1** - **6** in CDCl_3_ (500 MHz)

| **Pos.** | **1** | | | **2** | | | **3** | | | **4** | | **5** | | **6** | |
| --- | --- | --- | --- | --- | --- | --- | --- | --- | --- | --- | --- | --- | --- | --- | --- |
|  | **^1^H NMR**  **δ, mult., J(Hz)** | **^13^C NMR** | **HMBC** | **^1^H NMR** | **^13^C NMR** | **HMBC** | **^1^H NMR** | **^13^C NMR** | **HMBC** | **^1^H NMR** | **^13^C NMR** | **^1^H NMR** | **^13^C NMR** | **^1^H NMR** | **^13^C NMR** |
| 1 | 4.21 t, (5.5) | 59.2 (CH_2_) | C-2, C-3 | 4.48, d, (5.0) | 59.2 (CH_2_) | C-3, C-4 | 4.85, d, (5.0 ) | 61.3 (CH_2_) | C-3′ | 5.55 d, (16.6)  5.34 d, (11.2) | 116.8 (CH_2_) | 5.55 d, (17.5)  5.33 d, (10.4) | 116.3 (CH_2_) | 5.23, d, (10.5)  5.24, d (17.4) | 115.7 (CH2) |
| 2 | 5.54 t, (6.5) | 128.6 (CH) | C-1, C-4, C-15 | 6.78, d, (10.5) | 140.8 (CH) | C-4 | 6.6, t, (4.8) | 139.1 (CH) | C-15, C-4 | 6.02 dd, (11.0, 17.0) | 139.6 (CH) | 5.99 dd, (11.0, 17.0) | 139.3 (CH) | 5.89, dd, (10.5, 17.0) | 144.3 (CH) |
| 3 |  | 135.1 (C) |  |  | 134.7 (C) |  |  | 135.3 (C) |  |  | 80.2 (C) |  | 80.1 (C) |  | 69.4 (C) |
| 4 | 4.51 dd, (11.2, 2.5) | 86.9 (CH) | C-2, C-3, C-15 |  | 203.1 (C) |  |  | 201.6 (C) |  |  | 212.2 (C=O) |  | 212.2 (C=O) | 3.32, dd, (10.5, 11.0) | 90.2 (CH) |
| 5a | 1.92 ddd, (13, 13, 11) | 26.1. (CH_2_) | C-3, C-4, C-6, C-7 | 2.95, d, (11.0, 3.0) | 33.5 (CH_2_) | C-3, C-7, C-11 | 2.93 dd, (6.0) | 34.0 (CH_2_) | C-7, C-11 | 2.85 m | 32.8 (CH2) | 2.78, m | 32.4 (CH2) | 1.27, dd (11.0, 3.0) |  |
| 5b | 1.66 dt, (13, 3) |  | C-6, C-7 | 1.79, d, (13.0, 3.0) |  | C-3, C-7, C-11 |  |  |  | 2.76 m |  | 2.65, m |  | 1.52, dd (11.0, 3.0) |  |
| 6 | 2.41 dd, (13.0, 3.3) | 49.7 (CH) | C-5, C-7, C-11, C-12, C-13 | 2.80 dd, (13.0, 3.0) | 48.9 (CH) | C-5, C-7, C-10, C-11 | 2.85 t, (6.0) | 49.4 (CH) | C-5, C-7, C-11, C-14 | 2.85 m | 49.0 (CH) | 3.01, m | 49.2 (CH) | 1.89, dd, (10.4, 11.3) | 46.4 (CH) |
| 7 |  | 79.6 (C) |  |  | 70.9 (C) |  |  | 71.1 (C) |  |  | 71.4 (C) |  | 71.3 (C) |  | 71.9 (C) |
| 8 | 6.54 d, (10.2 ) | 150.6 (CH) | C-6, C-10 | 6.78, d, (10.5) | 155.3 (CH) | C-6, C-10 | 6.76 d, (10.2) | 155.6 (CH) | C-6, C-7, C-10, C-14 | 6.80 d (10.3) | 155.4 (CH) | 6.80, d (10.3) | 155.2 (CH) | 6.78, d (10.4) | 146.9 (CH) |
| 9 | 5.58d, (10.5) | 128.1 (CH) | C-7, C-11 | 5.84, d, (10.5) | 124.9 (CH) | C-7, C-11 | 5.78 d, (10.2) | 125.2 (CH) | C-7, C-11 | 5.90 d (10.3) | 125.6 (CH) | 5.90, d (10.3) | 125.4 (CH) | 6.13, d, (10.4) | 126.1 |
| 10 |  | 203.6 (C) |  |  | 203.1 (C) |  |  | 203.2 (C) |  |  | 202.7 (C=O) |  | 202.7 (C=O) |  | 203.0 (C=O) |
| 11 |  | 43.7 (C) |  |  | 45.2 (C) |  |  | 45.5 (C) |  |  | 45.5 (C) |  | 45.2 (C) |  | 45.2 (C) |
| 12 | 1.05 s | 20.8 (CH_3_) | C-6, C-10, C-11, C-13 | 1.08, s | 21.2 (CH_3_) | C-6, C-11, C-13 | 1.12, s | 24.9 (CH_3_) | C-6, C-11, C-13, | 1.06 s | 25.4 (CH_3_) | 1.12 s | 25.2 (CH_3_) | 1.26, s | 21.8 (CH_3_) |
| 13 | 1.15 s | 25.9 (CH_3_) | C-6, C-10, C-11, C-13 | 1.08, s | 24.5 (CH_3_) | C-6, C-11, C-13 | 1.12, s | 14.3 (CH_3_) | C-6, C-11, C-12, | 1.06 s | 23.1 (CH_3_) | 1.06 s | 22.9 (CH_3_) | 1.26, s | 23.8 (CH_3_) |
| 14 | 1.55 s | 21.4 (CH_3_) | C-6, C-7, C-8 | 1.32, s | 22.9 (CH_3_) |  | 1.31, s | 23.3 (CH_3_) | C-6 | 1.34 s | 23.1 (CH_3_) | 1.34 s | 23.2 (CH_3_) | 1.35, s | 21.5 (CH_3_) |
| 15 | 1.74 s | 13.9 (CH_3_) | C-2, C-3, C-4 | 1.89, s | 11.9 (CH3) |  | 1.81, s | 12.5 (CH_3_) | C-2, C-4 | 1.54 s | 25.2 (CH_3_) | 1.53 s | 25.2 (CH_3_) | 1.71, s | 23.7 (CH_3_) |
| 1’ |  |  |  |  |  |  |  | 173.8 (C) |  |  |  |  |  |  |  |
| 2’ |  |  |  |  |  |  | 2.33 t, (5.0) | 34.3 (CH_2_) |  |  |  |  |  |  |  |
| 3’ |  |  |  |  |  |  | 1.25, m | 31.8 (CH_2_) |  |  |  |  |  |  |  |
| 4’-9’ |  |  |  |  |  |  | 1.25, m | 30.5 (6CH_2_) |  |  |  |  |  |  |  |
| 10’ |  |  |  |  |  |  | 0.82, t | 14.3 (CH3) |  |  |  |  |  |  |  |
